# The Cholinesterase Inhibitory Properties of Stephaniae Tetrandrae Radix

**DOI:** 10.3390/molecules25245914

**Published:** 2020-12-14

**Authors:** Xiang-Peng Kong, Hai-Qin Ren, Etta Y. L. Liu, Ka-Wing Leung, Shu-Chen Guo, Ran Duan, Tina T. X. Dong, Karl W. K. Tsim

**Affiliations:** 1 Institute of Pharmaceutical & Food Engineering, Chinese Medicine Master Studio of Wang Shimin, Shanxi University of Chinese Medicine, 121 Daxue Road, Yuci District, Jinzhong 030619, China; xpkong@ust.hk; 2 Shenzhen Key Laboratory of Edible and Medicinal Bioresources, HKUST Shenzhen Research Institute, Hi-Tech Park, Shenzhen 518057, China; haiqinren@163.com (H.-Q.R.); lkwing@ust.hk (K.-W.L.); scgou@ust.hk (S.-C.G.); duanran@ust.hk (R.D.); botina@ust.hk (T.T.X.D.); 3 Division of Life Science and Center for Chinese Medicine, The Hong Kong University of Science and Technology, Clear Water Bay, Hong Kong, China; yliuch@connect.ust.hk; 4 Key Laboratory of Food Quality and Safety of Guangdong Province, College of Food Science, South China Agricultural University, Guangzhou 510642, China

**Keywords:** cholinesterase, Stephaniae tetrandrae radix, alkaloid, fangchinoline

## Abstract

Stephaniae tetrandrae radix (STR) is a commonly used traditional Chinese medicine in alleviating edema by inducing diuresis. In the clinic, STR extracts or its components are widely used in the treatment of edema, dysuria, and rheumatism for the regulation of water metabolism. Furthermore, STR has been used in treating emotional problems for years by combining with other Chinese herbs. However, the material basis and mechanism of STR on the nervous system have not been revealed. Here, the main components of STR extracts with different extracting solvents were identified, including three major alkaloids, i.e., cyclanoline, fangchinoline, and tetrandrine. The cholinesterase inhibitory activity of STR extracts and its alkaloids was determined using the Ellman assay. Both cyclanoline and fangchinoline showed acetylcholinesterase (AChE) inhibitory activity, demonstrating noncompetitive enzyme inhibition. In contrast, tetrandrine did not show enzymatic inhibition. The synergism of STR alkaloids with huperzine A or donepezil was calculated by the median-effect principle. The drug combination of fangchinoline–huperzine A or donepezil synergistically inhibited AChE, having a combination index (CI) < 1 at *F_a_* = 0.5. Furthermore, the molecular docking results showed that fangchinoline bound with AChE residues in the peripheral anionic site, and cyclanoline bound with AChE residues in the peripheral anionic site, anionic site, and catalytic site. In parallel, cyclanoline bound with butyrylcholinesterase (BChE) residues in the anionic site, catalytic site, and aromatic site. The results support that fangchinoline and cyclanoline, alkaloids derived from STR, could account for the anti-AChE function of STR. Thus, STR extract or its alkaloids may potentially be developed as a therapeutic strategy for Alzheimer’s patients.

## 1. Introduction

Alzheimer’s disease (AD) is a neurodegenerative disorder characterized by memory loss, cognitive impairment, and behavioral disturbances [1]; the pathological manifestations, e.g., brain neuronal loss, extracellular amyloid β-peptide (Aβ) deposition, neurofibrillary tangles, and neuroinflammation typically occur simultaneously [2,3,4]. The cholinergic system is critically important for brain function in cognition and neuroinflammation, and the decrease in cholinergic neurotransmission is closely associated with symptoms of dementia [5]. Acetylcholinesterase (AChE) and butyrylcholinesterase (BChE) are the enzymes responsible for cholinergic neurotransmission. AChE is the crucial enzyme in the nervous system in regulating neurotransmission at the cholinergic synapse by rapidly hydrolyzing neurotransmitter acetylcholine (ACh). BChE is an enzyme which hydrolyzes the non-natural substrate butyrylcholine; however, its biological role remains obscure. AChE is a widely used therapeutic target in AD treatment, and it is an effective strategy to inhibit enzymatic activity, as well as restore the depleted ACh in AD brain [6]. Indeed, AChE inhibitors, e.g., tacrine, rivastigmine, and donepezil, are employed to improve cholinergic transmission in the brain to slow the progression of AD [7,8,9]. Due to the complexity of AD pathogenesis, most of these drugs suffer from toxic effects to a certain degree, and precise and efficient treatments are still lacking [10,11,12].

Recent studies suggested that inflammatory response plays an essential role in the development of AD [13,14]. In identifying the relationship between AD and inflammation, the incidence of AD in populations suffering from rheumatoid arthritis was found to be significantly higher than that in the normal population, and the risk of AD was found to be prevalent in the case of chronic inflammatory diseases among rheumatoid arthritis patients [15]. Thus, anti-inflammatory therapy has been proposed to reduce the risk of developing dementia [16]. Traditional Chinese medicines (TCMs), e.g., Coptidis rhizoma (the rhizome of *Coptis chinensis* Franch.), Lycopodii herba (the whole herb of *Lycopodium japonicum* Thunb.), and Zingiberis rhizoma recens (the rhizome of *Zingiber officinale* Rosc.), have shown good potency in treating rheumatic disease for their properties in eliminating dampness or dispelling rheumatism [17,18,19]. Interestingly, these TCMs demonstrated better AChE inhibitory and immune regulatory activities, with comprehensive therapeutic effects in improving cognitive activities. Therefore, this should be an efficient basis to screen leading ingredients for AD treatment from anti-inflammatory or AChE inhibitory TCM products [20,21]. Stephaniae tetrandrae radix (STR; the root of *Stephania tetrandra* S. Moore) is a common TCM herb in treating edema, dysuria, and rheumatism. The main components of STR are alkaloids, i.e., tetrandrine and fangchinoline, which show beneficial effects on brain disease by regulating the synthesis, expression, and metabolism of neurotransmitters [22,23,24,25]. However, there are very few systematic studies on the cholinesterase regulation of STR, as well as its active components. Here, the cholinesterase inhibitory properties of STR and its major alkaloids were measured, and the synergistic effect of STR alkaloids with huperzine A or donepezil was investigated.

## 2. Results

### 2.1. Determination of Alkaloids in STR Extracts

**Figure 1:**
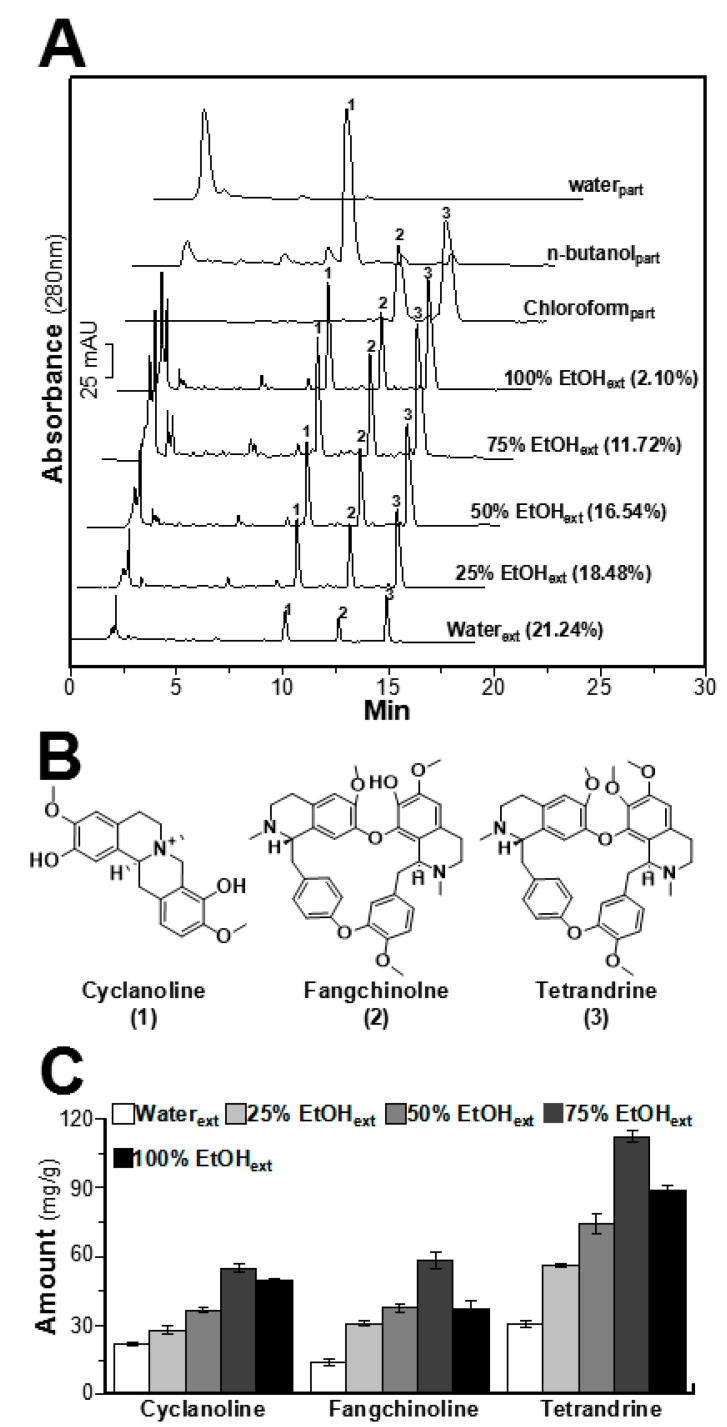
The HPLC chromatograms of different Stephaniae tetrandrae radix (STR) extracts and structures of STR alkaloids. (**A**) The profiles of STR extracts are shown, and the *x*-axis and *y*-axis represent retention time (min) and absorbance units (mAU) at 280 nm, repectively. The yields of STR extracts using water and different fractions of ethanol (25%, 50%, 75%, and 100%) are listed in parentheses beside the chromatogram. The main alkaloids of STR extracts were identified using known chemical standards. The identification numbers of alkaloids in STR extracts are labeled in HPLC chromatograms as follows: 1, cyclanoline; 2, fangchinoline; 3, tetrandrine. (**B**) Structures of alkaloids in STR extracts. The structures are numbered and listed from 1 to 3 according to the HPLC in (**A**). (**C**) Amounts of alkaloids in STR extracts using different solvents according to HPLC analysis, as in (**A**). Values are the mean ± SEM (standard error of mean) of three dependent determinations.

The HPLC chromatograms of STR extracts using different volume fractions of ethanol are shown in Figure 1A. Except for the STR extract with 100% ethanol, the contents of cyclanoline, fangchinoline, and tetrandrine in STR extracts increased obviously upon increasing the ethanol fraction in the solvent. The structures of these alkaloids are shown in Figure 1B. However, the yields of STR extracts decreased upon increasing the ethanol fraction in the solvent (Figure 1A). As shown from the HPLC chromatograms using chloroform, *n*-butanol, and water for extraction of STR based on polarity in Figure 1A, fangchinoline and tetrandrine were the major alkaloids in the chloroform extract of STR. In parallel, cyclanoline was the principal alkaloid in the *n*-butanol extract of STR. Furthermore, the amounts of individual alkaloids in STR crude drug were determined, and the linear relationships (*R*^2^ of each component > 0.999) were calibrated from the calibration curve (Table 1). The maximal amount of STR alkaloids was identified in the solvent with 75% ethanol, i.e., accounting for about 220 mg of total alkaloids in 1 g of dried herbal extract (Figure 1C). Tetrandrine was the most abundant alkaloid in STR extracts, particularly in solvents with high ethanol content. Cyclanoline and fangchinoline were present in roughly equal amounts in the STR extracts.

### 2.2. STR Extracts Inhibit Cholinesterase Activity

**Figure 2:**
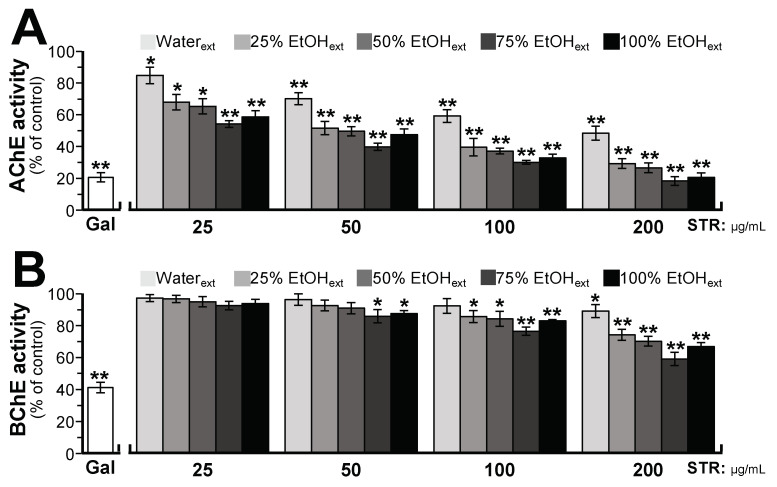
The water and ethanol extracts of STR inhibit cholinesterase activity. STR extracts using water and different fractions of ethanol (25%, 50%, 75%, and 100%) were tested for acetylcholinesterase (AChE) activity (**A**) and butyrylcholinesterase (BChE) activity (**B**). Galanthamine (Gal, 10 or 60 μM) served as a positive control for AChE or BChE activity. Data are expressed as the mean ± SEM (standard error of mean) of three dependent assays, each with triplicate samples. * *p* < 0.05 and ** *p* < 0.01 compared to control (no drug).

STR extracts using water and different fractions of ethanol (25%, 50%, 75%, and 100%) were tested for AChE inhibitory activity using the Ellman assay. As shown in Figure 2, the inhibitory activities of STR extracts on AChE and BChE increased in accordance with the amount of ethanol in the extracting solvent. Beyond 75% ethanol, the extract showed maximal inhibition on AChE and BChE, accounting for ~82% and ~41% inhibition at the dose of 200 μg/mL. Furthermore, the anti-AChE or anti-BChE activities correlated with the amounts of alkaloids within the STR extracts. Therefore, the alkaloids could represent the major cholinesterase inhibitory components in STR extracts. In addition, the STR alkaloids showed more potency on AChE than BChE, i.e., approximately twofold greater inhibition of AChE than BChE.

**Figure 3:**
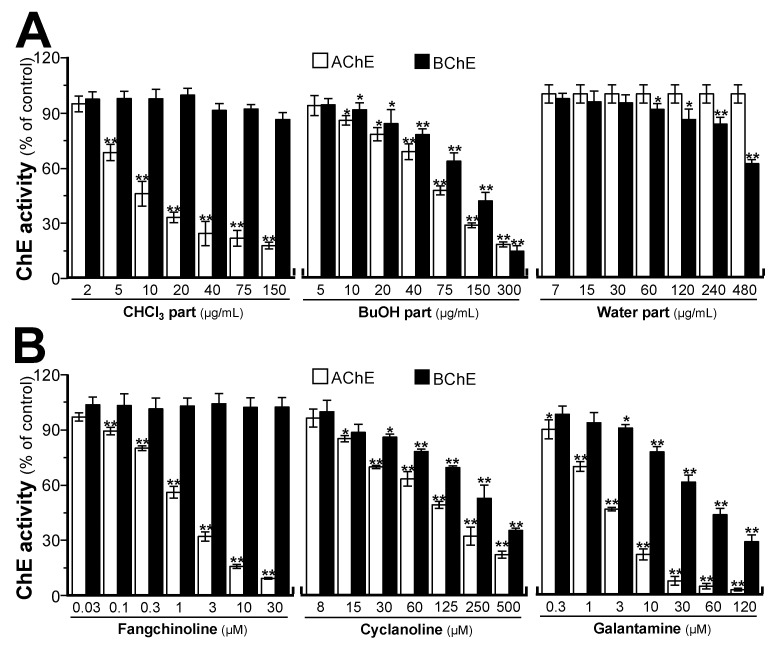
The extracts of STR and its main alkaloids inhibit cholinesterase activity. (**A**) STR extracts using different solvents inhibited cholinesterase activity. (**B**) STR alkaloids and galanthamine inhibited cholinesterase activity. Data are expressed as the mean ± SEM (standard error of mean) of three dependent assays, each with triplicate samples. * *p* < 0.05 and ** *p* < 0.01 compared to control.

The different polar extracts (chloroform, *n*-butanol, and water) of STR were prepared and tested for AChE and BChE activities. As shown in Figure 3A, the chloroform extract showed a good dose–effect relationship with AChE inhibition, i.e., inhibiting AChE activity with a half maximal inhibitory concentration (IC_50_) of 11.82 ± 2.25 μg/mL, while it showed no inhibitory activity on BChE. The *n*-butanol extract showed a good dose–effect relationship with AChE and BChE inhibition with IC_50_ values of 69.13 ± 4.41 and 108.35 ± 5.16 μg/mL, respectively. However, the water extract showed no AChE inhibitory activity and a weak inhibitory activity on BChE at high concentration. The chloroform extract of STR mainly contained bisbenzylisoquinoline alkaloids, including fangchinoline and tetrandrine, while the *n*-butanol extract of STR mainly contained cyclanoline. The water extract contained much lower levels of these alkaloids. Therefore, it was speculated that the above alkaloids were closely related to the potency of STR in terms of cholinesterase inhibition.

**Figure 4:**
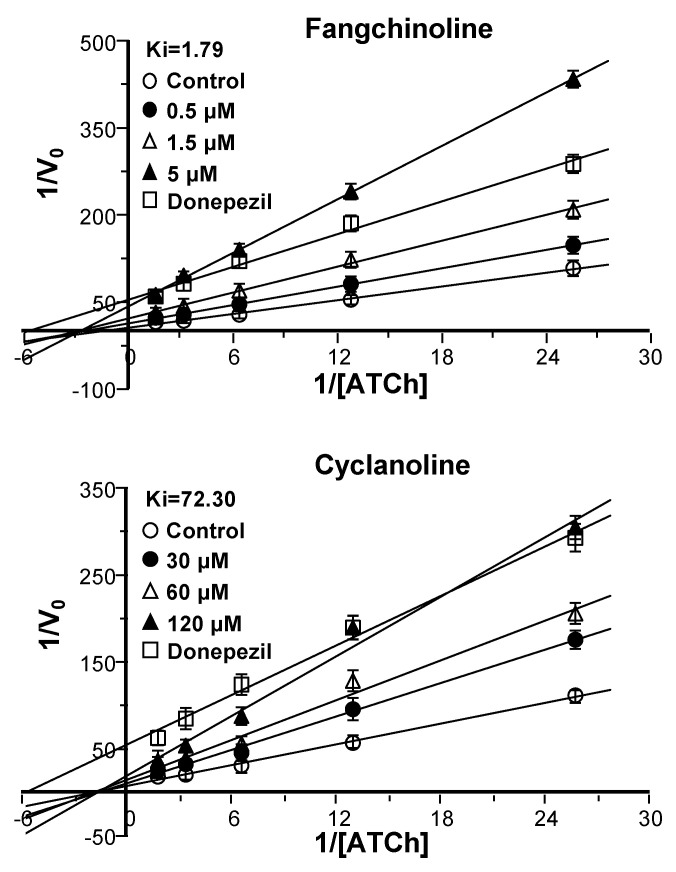
The enzyme kinetic analysis of AChE inhibition by fangchinoline and cyclanoline. Different doses of fangchinoline (0.5–5 μM) and cyclanoline (30–120 μM) were added to the AChE reaction system and preincubated at 37 °C with the brain lysate, followed by the addition of a range of doses of ATCh (0.040–0.625 mM). The inhibition constants *K_i_* were estimated from the plots of the slope versus the concentration of tested drugs. Donepezil (25 nM) served as a positive control. The data are expressed as the mean ± SEM (standard error of mean) of three dependent assays, each with triplicate samples.

To reveal the material basis of STR extracts on AChE and BChE inhibitory activities, the major alkaloids in different polar extracts (chloroform, *n*-butanol, and water) of STR were further tested. As shown in Figure 3B, fangchinoline showed vigorous inhibitory activity on AChE with an IC_50_ of 2.58 ± 0.28 μM, while it demonstrated no inhibitory activity on BChE. In parallel, its structural analogue tetrandrine exhibited no inhibitory activity on AChE at the maximal dissolvable concentration (data was not shown), as well as no or little inhibitory activity on BChE. However, cyclanoline exhibited weak AChE and BChE inhibitory activities with IC_50_ values of 105.6 ± 2.29 and 246.65 ± 5.46 μM, respectively. Galanthamine served as a positive control, showing inhibitory activity on AChE and BChE with IC_50_ values of 2.46 ± 0.17 and 49.82 ± 2.50 μM, respectively. In addition, the kinetics of AChE inhibition by fangchinoline and cyclanoline were studied. Serial concentrations of fangchinoline (0.5–5 μM) and cyclanoline (30–120 μM) were added to AChE lysate containing a range of acetylthiocholine iodide (ATCh, 0.040–0.625 mM). As determined from the Lineweaver–Burk plots, the *K_i_* values were estimated as 1.79 mM for fangchinoline and 72.30 mM for cyclanoline (Figure 4). The inhibition of fangchinoline and cyclanoline in AChE was noncompetitive as the *K_m_* of fangchinoline and cyclanoline did not change from 0.345 ± 0.021 and 0.557 ± 0.044 mM, respectively.

### 2.3. Synergy of Fangchinoline and Donepezil or Huperzine A

**Figure 5:**
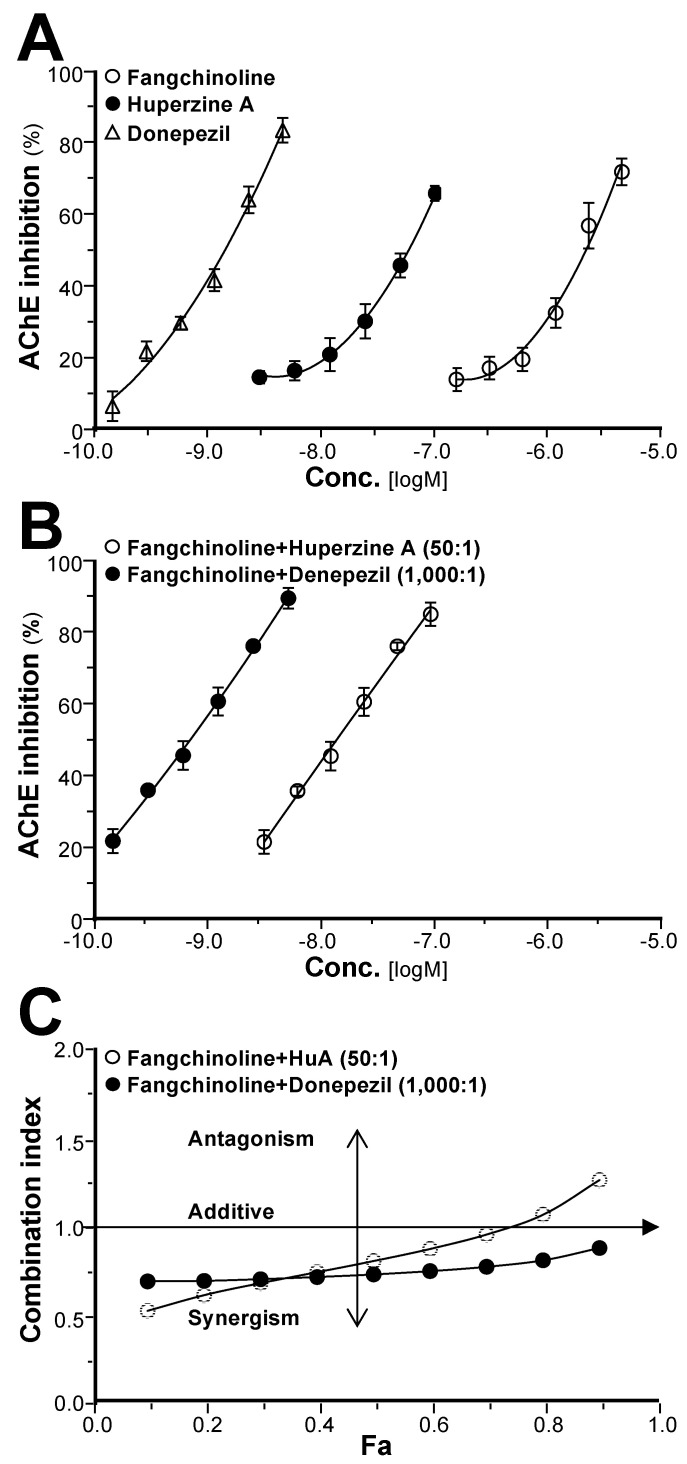
Synergy of fangchinoline and huperzine A or donepezil on AChE inhibition. (**A**) Fangchinoline (0.156–5 μM), huperzine A (3.125–100 nM), and donepezine (0.156–5 nM) dose-dependently inhibited AChE. (**B**) Fangchinoline and huperzine A or donepezil dose-dependently inhibited AChE. Different doses of fangchinoline, huperzine A, donepezil, and fangchinoline–huperzine A or fangchinoline–donepezil combinations were added to the assay solution and preincubated at 37 °C with the brain lysate, followed by the addition of ATCh (0.625 mM). The absorbance of the assay solution at 405 nm was measured. The data were calculated as the percentage inhibition, and the results are expressed as the mean ± SEM (standard error of mean) of three dependent assays, each with triplicate samples. (**C**) Synergism analysis of fangchinoline and huperzine A or donepezil evaluated by the median-effect principle. The combination index (CI) values of these combinations were calculated as described in Section 4. The *F_a_*–CI plots of fangchinoline and huperzine A or donepezil are demonstrated. Values of CI < 1, CI = 1, and CI > 1 refer to synergistic, additive, and antagonistic effects, respectively.

Considering the compatibility theory of drugs, fangchinoline and the represented compound huperzine A or donepezil were tested for their synergistic effect on AChE inhibition. Fangchinoline, huperzine A, and donepezil inhibited AChE in dose-dependent manners, having IC_50_ values of 2.12 ± 0.16 μM, 56.10 ± 2.04 nM, and 1.44 ± 0.07 nM, respectively (Figure 5A). The combinations of fangchinoline and huperzine A or donepezil (with concentration ratios of 50:1 or 1000:1, respectively), showed better AChE inhibition in dose-dependent manners (Figure 5B). In addition, the inhibitory activity of fangchinoline and huperzine A or donepezil on AChE increased substantially, with IC_50_ values (expressed as huperzine A and donepezil) of 28.93 ± 1.41 nM and 0.68 ± 0.08 nM, respectively, which were much lower than when using their single drug alone. The synergistic effects of fangchinoline and huperzine A or donepezil on AChE inhibition were further evaluated by the median-effect principle. As shown from the *F_a_*–combination index (CI) plot in Figure 5C, when *F_a_* = 0.5, the CI values of the fangchinoline and huperzine A or donepezil combination (at the ratios of 50:1 or 1000:1, respectively) were 0.80 and 0.72, respectively, suggesting a synergistic effect. However, the CI value of the fangchinoline and huperzine A combination increased with increasing dose or *F_a_* value, which indicated that the synergy decreased as a function of the dose. When *F_a_* > 0.7, the CI value of fangchinoline and huperzine A was close to or greater than one, indicating that the inhibitory effect of their combination might be additive or antagonistic. In parallel, the synergistic effect of fangchinoline and donepezil on AChE slowly decreased with increasing dose, while the CI value of the combination was less than one at different *F_a_* values.

### 2.4. Cholinesterase Binding Site Analysis of STR Alkaloids

**Figure 6:**
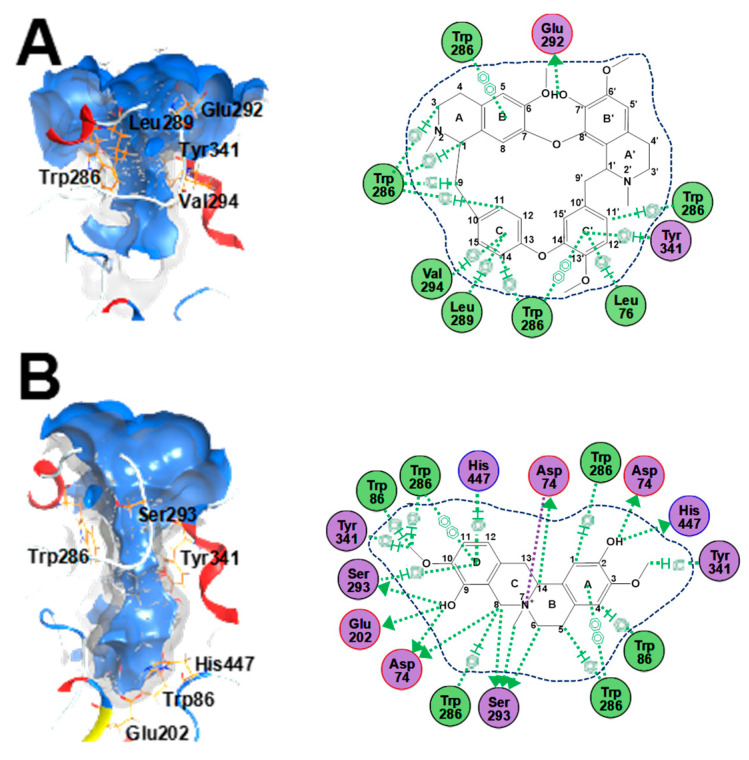
The binding modes of fangchinoline and cyclanoline with AChE (Protein Data Bank (PDB) code: 4EY7). (**A**) The three-dimensional (3D) surfaces and interaction maps of fangchinoline with AChE (left) and the 2D interaction of fangchinoline with AChE (right). (**B**) The 3D surfaces and interaction maps of cyclanoline with AChE (left) and the 2D interaction of cyclanoline with AChE (right). The dotted arrow from the structure to amino-acid residues represents the sidechain donor. The arene symbol represents π bond interactions with other chemical groups.

The active site of AChE is a deep and narrow gorge (Figure 6). The binding modes of fangchinoline and cyclanoline with AChE (Protein Data Bank (PDB) code: 4EY7) were different in terms of their interactions with the amino-acid residues, i.e., fangchinoline mainly bound with the peripheral anionic site at the top of the AChE active pocket, while cylanoline bound with the peripheral anionic site, anionic site, and catalytic site along the gorge pocket. As shown in Table 2, the fitting scores of fangchinoline and cyclanoline were rather similar at −7.11 ± 0.32 and −7.00 ± 0.01, respectively. There was a π–π interaction between the aromatic ring (B and C′) of fangchinoline and Trp-286 (peripheral anionic site) of the AChE protein with a binding affinity of −35.14 kJ/mol, and an H–π interaction between hydrogen atoms in the hydrocarbon groups (1, 3, 9, 11, 14, and 11′) of fangchinoline and Trp-286 with a binding affinity of −35.85 kJ/mol (Figure 6A). Furthermore, there was a π–H interaction between the aromatic ring (C and C′) of fangchinoline and AChE residues of Leu-76, Leu-289, Val-294, and Tyr-341 with binding affinities of −2.74, −3.34, −0.70, and −2.41 kJ/mol, respectively. There was also an H–donor interaction between hydrogen atoms (7′-OH) of fangchinoline and the AChE residue of Glu292 with a binding affinity of −5.68 kJ/mol. In parallel, the binding mode of tetradrine with AChE was analyzed (data are not shown); there were π–π and H–π interactions between tetrandrine (via the aromatic ring and hydrogen atoms in methoxy, methene, and aromatic groups) and Trp-286/Tyr-72 with binding affinities of −24.69, −17.11, and −3.13 kJ/mol, much higher than seen for fangchinoline. Meanwhile, there was a π–H interaction between the aromatic ring (B′ and C′) of tetrandrine and AChE residues of Leu-76 and Glu-292, where the binding affinity with Glu-292 was also higher than seen with fangchinoline. Furthermore, there was an H–acceptor interaction between the nitrogen atom (2, 2′) of tetrandrine and Val-294 with a binding affinity of −7.13 kJ/mol. Therefore, it could be speculated that the binding mode of fangchinoline with the AChE peripheral anionic site was more stable than that of tetrandrine, and the residues of Trp-286, Tyr-341, and Glu-292 contributed to their AChE inhibitory activities.

As for cyclanoline, there were similar binding targets with the peripheral anionic site (Figure 6B), i.e., π–π and H–π interactions with Trp-286 via the aromatic ring (A and D) and hydrogen atoms in the aromatic ring, methene, and methoxy groups (1, 5, 8, and 10), with binding affinities of −33.24 and −45.78 kJ/mol, respectively, the H–π interaction with Tyr-341 via hydrogen atoms in hydrocarbon bond groups (3 and 10) with a binding affinity of −1.95 kJ/mol, and metal/ion and H–donor interactions with Asp-74 via the nitrogen atom (7) and hydrogen atoms in hydroxyl (2 and 9), methene, and methine groups (8 and 14) with binding affinities of −2.64 and −8.95 kJ/mol, respectively. Cyclanoline also bound with the catalytic site, i.e., via an H–donor interaction with His-447 via hydrogen atoms (2-OH) with a binding affinity of −1.33 kJ/mol and a π–H interaction with His-447 via the aromatic ring (D) with a binding affinity of −1.65 kJ/mol. Furthermore, there was an H–π interaction with Trp-86 (the anionic site) via hydrogen atoms in the aromatic ring and methoxy groups (4 and 10) of cyclanoline with a binding affinity of −4.92 kJ/mol. There was also an H–donor interaction with Ser-293 and Glu-202 via hydrogen atoms in hydroxyl, methylene, and nitromethyl groups with binding affinities of −10.99 and −3.73 kJ/mol, respectively. As revealed from target analysis, cyclanoline could bind with AChE residues in the peripheral anionic site, catalytic site, and anionic site, as well as with other residues along with AChE gorge pocket, and it could be determined that the residues of Trp-286, Asp-74, His-447, Trp-86, and Ser-293 contributed to the AChE inhibitory activity of cyclanoline.

**Figure 7:**
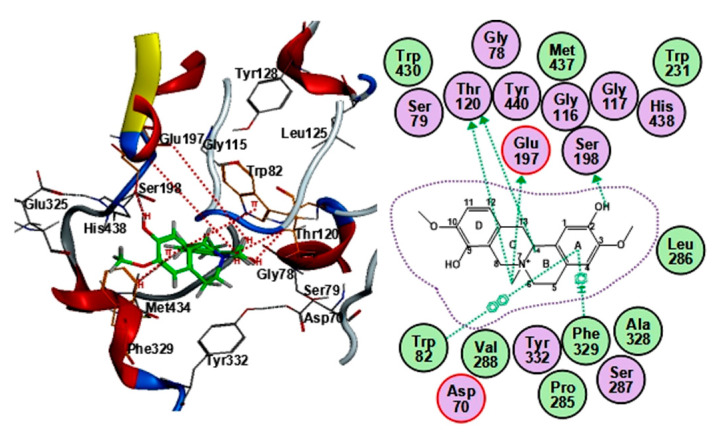
The binding mode of cyclanoline with BChE (PDB code: 4BDS). The 3D (left) and 2D (right) interaction of cyclanoline with BChE. The ligand is shown in green in the 3D interaction diagram. The dotted arrow from the structure to amino-acid residues represents the sidechain donor. The arene symbol represents π bond interactions with other chemical groups.

It was previously shown that BChE shares ~55% sequence homology with AChE [26], and the differences in amino-acid residues between BChE and AChE lead to their selectivity for ligands and substrates [27]. In parallel, the BChE binding mode of cyclanoline was analyzed (Figure 7). The fitting mode of cyclanoline with BChE was significantly different from the binding mode with AChE: (i) interaction with Ser-198 via an H–donor bond in the catalytic triad consisting of Ser-198, Glu-325, and His-438 (corresponding to Ser-200, Glu-334, and His-447 in AChE); (ii) interaction with Trp-82 via a π–π bond in the anionic site (corresponding to Trp-86 in AChE); (iii) interaction with Phe-329 via a π–H bond in the aryl hydrophobic pocket consisting of aromatic residues (corresponding to Phe-331 in AChE); (iv) interaction with Glu-197 and Thr-120 (corresponding to Glu-199 and Ser-122 in AChE). The fitting score of cyclanoline was −6.68 ± 0.20, higher than that of AChE. Furthermore, there are more or higher binding residues in the peripheral anionic site and anionic site of AChE than BChE, which leads to the binding difference of BChE and AChE. There were only interactions with the peripheral anionic and anionic sites in terms of fitting modes of fangchinoline and tetrandrine with BChE (data not shown), e.g., involving Asp-70, Tyr-332, and Tyr-82, and neither of them could bind with the catalytic site. The results suggested that interactions with residues in the catalytic site may at least contribute to an inhibitory activity on BChE.

## 3. Discussion

AD is a complex neurodegenerative disease leading to impaired cognition [1,4,5,28], and drugs targeting AChE represent the primary treatment method in the modern clinic. TCMs have been used in treating mental and emotional problems for years, and the TCM prescription Fangji Dihuang Tang (described in “Jingui Yaolve”) has been used in treating emotional diseases for years [29,30]. Our preliminary study showed that the alkaloid-containing herbal medicine STR in Fangji Dihuang Tang has a certain degree of AChE inhibition [22,29]. To reveal the material basis of STR on cholinesterase, the cholinesterase inhibitory properties of STR, as well as its main active ingredients, were studied systematically. As shown from the determination of the main active ingredients of STR, the bisbenzylisoquinoline (lipid-soluble alkaloids, e.g., fangchinoline and tetrandrine) and protoberberine (water-soluble alkaloids, e.g., cyclanoline) alkaloids were the two main types of alkaloid (Figure 1). Furthermore, the AChE and BChE inhibitory activities of STR extracts using water and different fractions of ethanol increased in accordance with the alkaloid amount (Figure 2). Further study showed that the bisbenzylisoquinoline alkaloids inhibited AChE and the water-soluble protoberberine alkaloids inhibited both AChE and BChE activities (Figure 3). Fangchinoline was the representative bisbenzylisoquinoline alkaloid accounting for the efficacy of AChE inhibition, while its structural analogue, tetrandrine, did not have AChE inhibitory activity. Cyclanoline was the main proberberine alkaloid, and its inhibitory activity of AChE was comparable to that of BChE. Both fangchinoline and cyclanoline showed similar noncompetitive AChE inhibition (Figure 4). The *K_i_* value of fangchinoline was much lower than that of cyclanoline. Through molecular docking analysis, the binding modes of fangchinoline, tetrandrine, and cyclanoline with AChE or BChE were found to be different (Figure 6). The interaction of fangchinoline with Trp-286/Tyr-72 in the peripheral anionic site was more potent than that of tetrandrine, which could, in part, account for their different inhibition of AChE. The comparative simulation study of fangchinoline and cyclanoline showed that the latter could bind with the peripheral anionic site, anionic site, and catalytic site, with an overall interaction pattern superior to that of fangchinoline. Therefore, it could be speculated that other factors, e.g., spatial effect, hydrophobic interaction force, or polarity, may be responsible for the difference in inhibitory activity of AChE. In terms of binding mode with BChE, the interactions of cyclanoline with residues in the peripheral anionic site, anionic site, and catalytic site were weaker than those with AChE. Meanwhile, the comparative investigation between cyclanoline and fangchinoline or tetrandrine demonstrated that the catalytic site residue Ser-198 may contribute to the inhibitory activity of cyclanoline on BChE (Figure 7). Therefore, the above results support that alkaloids are the main active ingredients of STR contributing to AChE and BChE inhibition, in which fangchinoline mainly accounted for the inhibition of AChE, while cyclanoline accounted for the inhibition of BChE. Moreover, fangchinoline and huperzine A or donepezil showed good synergistic effects on AChE inhibition (Figure 5), which indicates that fangchinoline may be an efficacious and promising remedy in AD treatment.

## 4. Materials and Methods

### 4.1. Chemicals and STR Extract Preparation

Fangchinoline (Lot S090409-10, purity 99%), tetrandrine (Lot S090601-5, purity 99%), and galanthamine (Lot DST190720-176, purity 99%) were purchased from Chengdu Ruifensi Biotechnology (Chengdu, China); huperzine A (Lot S090409-10, purity 99%) and donepezil (Lot S090409-10, purity 99%) were purchased from Chengdu DeSiTe Biotechnology (Chengdu, China). STR is the root of *S. tetrandra* from Jiangxi, China. The authentication of herbs was performed by Prof. Xiangping Pei from Shanxi University of Chinese Medicine. The STR herb was weighed appropriately, and extracts were obtained twice (1:10 *w*/*v*, 40 min each time) by reflux with 0%, 25%, 50%, 75%, and 100% ethanol volume fractions. The two filtrates were combined, concentrated to a sticky state, and dried at low temperature.

In order to prepare different polar extracts of STR, appropriate amounts of STR herb were extracted with 75% ethanol, according to the operation described above. The concentrated solution was extracted three times using chloroform with a volume ratio of 1:1. The chloroform solution was combined, and the remaining solution was subjected to extraction three times using *n*-butanol with a volume ratio of 1:1. The chloroform, *n*-butanol, and remaining water solutions were concentrated to a sticky state and dried at low temperature as above. The STR crude drug solution was prepared according to the Chinese Pharmacopoeia (2020 edition, part 1).

### 4.2. Determination of Alkaloids in Different STR Extracts

STR extracts (with 0%, 25%, 50%, 75%, and 100% ethanol volume fractions) were weighed accurately and dissolved in 50% MeOH at a concentration of 2 mg/mL. Different polar extractives of STR with solvents of chloroform, *n*-butanol, and water were weighed and dissolved to 0.80, 1.50, and 1.80 mg/mL, respectively. The dissolved extracts were filtered using a 0.22 μm Millipore filter, and the filtrate was subsequently collected for HPLC determination. The standards of fangchinoline, tetrandrine, and cyclanoline were weighed accurately and dissolved to a stock solution of 1 mg/mL using MeOH. The mixed standard solutions of fangchinoline, tetrandrine, and cyclanoline (with final concentrations of 100, 200, and 250 μg/mL, respectively) were prepared by mixing different volumes of the stock solution. The mixed stock solutions were diluted to a series of working standards with MeOH for HPLC determination. The HPLC chromatographic analysis was performed using a Waters 2695 HPLC (Waters Technology (Shanghai) Co., Ltd., Shanghai, China) equipped with an ultraviolet–visible light (UV-Vis) photodiode array detector. The sample separation was achieved on an Innoval C_18_ column (4.6 × 250 mm, 5 µm) with a flow rate of 1.0 mL/min at 25 °C. The mobile phase of STR was composed of MeCN (A) and water (B, 0.1% phosphoric acid) using a gradient elution of 10–30% A at 0–20 min and 30–55% A at 20–25 min. The detection was performed at 280 nm. The injection volume was set at 10 µL.

### 4.3. Preparation of Different STR Extracts and Alkaloids for Cholinesterase Activity Assay

STR extracts (with 0%, 25%, 50%, 75%, and 100% ethanol volume fractions) were weighed accurately and dissolved to a stock solution with a concentration of 40 mg/mL using dimethyl sulfoxide (DMSO). Different polar extracts of STR with chloroform, *n*-butanol, and water were weighed accurately and dissolved to a stock solution with concentrations of 30, 60, and 90 mg/mL using DMSO, respectively. The different concentrations of STR extracts were prepared for the evaluation of cholinesterase inhibitory activity by diluting the initial stock concentrations. The standards of cyclanoline, fangchinoline, tetrandrine, galanthamine, huperzine A, and donepezil were weighed accurately and dissolved to stock solutions with concentrations of 100, 50, 10, 50, 25, and 25 mM using DMSO, respectively. Different concentrations of these alkaloids were prepared for the evaluation of cholinesterase inhibitory activity by diluting initial stock concentrations. The combinations of fangchinoline and huperzine A or donepezil at a concentration ratio of 50:1 or 1000:1, respectively, were individually prepared by mixing appropriate volumes of fangchinoline and huperzine A or donepezil stock solutions. After that, the stock solutions of fangchinoline and huperzine A or donepezil were diluted to six concentrations (with initial system concentrations of 5:0.1 or 5:0.005 μM, respectively) using DMSO.

### 4.4. Cholinesterase Activity Assay

The extracts and alkaloids of STR were tested for cholinesterase activity using a 96-well microtiter plate (Corning, NY, USA) with a final volume of 200 μL, according to the Ellman method [31,32]. The mouse brain was lysate was prepared by homogenizing with precooled low-salt lysis buffer (1:10 *w*/*v*; containing 1 mM ethylenediaminetetraacetic acid (EDTA) and ethylene glycol tetraacetic acid (EGTA), 0.5% triton X-100, 150 mM NaCl, 10 mM 4-(2-hydroxyethyl)-1-piperazineethanesulfonic acid (HEPES, pH 7.4), 50 μg/mL benzamidine HCL, 10 μg/mL aprotinin, leupeptin, and pepstatin) for the AChE activity assay. The AChE assay medium consisted of brain lysate (5 mg/mL) with testing drugs, 80 mM Na_2_HPO_4_ (pH 7.4), 0.1 mM tetraisopropylpyro-phosphoramide (*iso*-OMPA), 0.5 mM DTNB (5′-dithiobis (2-nitrobenzenoic acid)), and 0.625 mM ATCh. The mixture of brain lysate, Na_2_HPO_4_ buffer, and *iso*-OMPA solution was incubated at 37 °C for 10 min, and then the solutions of DNTB and ATCh were added. After re-incubating at 37 °C for 30 min, AChE activity was tested by determining the absorbance at 405 nm.

Mouse serum (1: 50 *v*/*v*, diluted with precooled low-salt lysis buffer) was prepared for the BChE activity assay. The BChE assay medium consisted of the same composition as the AChE assay medium, except that the brain lysate and *iso*-OMPA were replaced with diluted serum solution and 0.1 μM BW284c51. The mixture of diluted serum, Na_2_HPO_4_ buffer, and BW284c51 solution was incubated at 37 °C for 10 min, and then the solutions of DNTB and ATCh were added. After re-incubating at 37 °C for 30 min, the BChE activity was tested by determining the absorbance at 405 nm. The concentration of DMSO in the reaction system of AChE/BChE assay was controlled at 0.5%. The background contrast without brain lysate or serum was set to eliminate the color interference at 405 nm. The AChE/BChE inhibitory activity of drugs was calculated as a percentage of the control absorbance value, AChE/BChE inhibition (%) = (1 − absorbance with inhibitor/absorbance without inhibitor) × 100%. Galanthamine was used as a positive control. The protein concentrations were measured using the Bio-Rad Protein Assay kit (Hercules, CA, USA).

The Michaelis constant (*K_m_*), maximum velocity (*V_max_*), and inhibitory constant (*K_i_*) were used to evaluate the kinetics of fangchinoline and cyclanoline. Lineweaver–Burk plots were then plotted using initial velocities, from which the *K_m_* and *V_max_* values were calculated (the absolute values of horizontal and vertical intercepts were 1/*K_m_* and 1/*V_max_*, respectively). The slopes of reciprocal plots were then plotted against the doses of fangchinoline and cyclanoline, and *K_i_* values were determined as the absolute value of the *x*-axis intercept. Donepezil was used as a positive control. All experiments were performed in triplicate.

### 4.5. Synergistic Effect Analysis by Median-Effect Principle

The synergistic effect between fangchinoline and huperzine A or donepezil was calculated by the median-effect principle [22,33,34]. The median-effect equation (*F_a_*/*F_u_* = (*D*/*D_m_*)*^m^*) was used to calculate the dose of fangchinoline, huperzine A, and donepezil contributing to AChE-specific inhibition according to the dose–effect curve. For comparison, the median-effect equation was transformed to a linear equation (log(*F_a_*/*F_u_*) = *m*log*D* − *m*log*D_m_*) by taking the logarithm of both sides, where *D* is the drug dose, *D_m_* represents the dose at 50% potency, *F_a_* is the AChE inhibition expressed as a decimal fraction, *F_u_* represents the unaffected fraction (*F_u_* = 1 − *F_a_*), and *m* is the coefficient of the median-effect equation (slope of the linear equation). The linearity curves and equations of fangchinoline, huperzine A, and donepezil were constructed by plotting *m*log*D* versus log(*F_a_*/*F_u_*) at each dose effect. The *D_m_* of fangchinoline, huperzine A, and donepezil was calculated according to their equations. The combination index (CI) was used to evaluate the synergistic effect of fangchinoline and huperzine A or donepezil according to the Chou–Talalay equation (CI = *D*_1_/*D*_1*x*_ *+ D*_2_/*D*_2*x*_), where *D*_1_ and *D*_2_ are the doses of the single drug required to produce *F_a_* in their combination, and *D*_1*x*_ and *D*_2*x*_ are the doses of the single drug producing the same effect alone. The values of *D*_1_, *D*_2_, *D*_1*x*_, and *D*_2*x*_ at different doses (*F_a_*, 0.1–0.9) were similarly calculated according to the linear equation (log(*F_a_*/*F_u_*) = *m*log*D* − *m*log*D_m_*), and then the CI values of fangchinoline and huperzine A or donepezil were calculated. CI values <1, =1, and >1 indicated synergistic, additive, and antagonistic effects, respectively.

### 4.6. Molecular Docking

To reveal the underlying binding targets of STR active components with AChE or BChE, a molecular docking study was used to analyze the interactions of fangchinoline, tetrandrine, and cyclanoline with the related residues of AChE or BChE. The three-dimensional (3D) structures of AChE (PDB code: 4EY7) and BChE (PDB code: 4BDS) were obtained from the Protein Data Bank [35,36], and the structural issues (missing loops, broken bonds, etc.) were corrected using MOE software (Chemical Computing Group, Inc., Montreal, Canada) [37]. Next, hydrogens were added to AChE and BChE and partial charges were calculated using the Amber10: EHT forcefield [38]. The active pocket was established using the binding site of the co-crystallized AChE ligand E2020. The structures of fangchinoline, tetrandrine, and cyclanoline were built and converted to 3D structures with minimized energy. In the Dock module of MOE, the methods of placement and refinement were selected as triangle matcher and rigid receptor with evaluated scores of London dG (the London dG scoring function estimates the free energy of binding of the ligand from a given pose) and GBVI/WSA ΔG (The GBVI/WSA ΔG is a forcefield-based scoring function which estimates the free energy of binding of the ligand from a given pose), while the poses of placement and refinement were set as 180 and 30, respectively. The rigid molecular docking simulation between STR alkaloids and the residue site in the AChE active pocket was performed using the MOE Dock module [39], followed by conformational analysis, placement, initial scoring, refinement, pharmacophore constraints, and final scoring. After that, the binding modes and target interactions of fangchinoline, tetrandrine, and cyclanoline were summarized by analyzing all conformation interactions with AChE residues.

### 4.7. Statistical Analysis

Statistical tests were done with DPS 18.10 software (Hangzhou Ruifeng Information Technology Co., Ltd., Hangzhou, China), using the bioassay module [40]. Data were expressed as the mean ± SEM (standard error of mean) of three independent experiments, with triplicates of each experiment. The multiple drug effect analysis was used to evaluate the drug interaction according to the median-effect principle, as described in Section 4.5.

## 5. Conclusions

The chemical composition of natural plants and herbs represents the effective material basis of TCM. Here, we proposed that alkaloids are the main active ingredients of STR in AChE and BChE inhibition. The enzyme activity results showed that the AChE inhibitory activities of STR extracts increased in accordance with the alkaloid amount. Bisbenzylisoquinoline and protoberberine alkaloids were the two main types of alkaloids contributing to the AChE/BChE inhibitory activity of STR, whereby the bisbenzylisoquinoline alkaloids inhibited AChE and the water-soluble protoberberine alkaloids inhibited both AChE and BChE activity. Fangchinoline was the representative bisbenzylisoquinoline alkaloid accounting for AChE inhibition, while its structural analogue, tetrandrine, did not have AChE inhibitory activity. Cyclanoline was the main proberberine alkaloid, and its inhibitory activity of AChE was comparable to that of BChE. Furthermore, both fangchinoline and cyclanoline exhibited noncompetitive AChE inhibition. The *K_i_* value of fangchinoline was much lower than that of cyclanoline. This result was also supported by the molecular docking simulation. In addition, the combination of fangchinoline and huperzine A or donepezil showed a good synergistic effect on AChE inhibition.

## Figures and Tables

**Table 1:** The linearity curves and contents of main alkaloids in STR crude drug.

Alkaloids	Linear Equation	*R* ^2^	Linear Range (μg/mL)	Content (mg/g)
Cyclanoline	*y* = 7732.9*x* + 21862	0.9997	7.8125–250	2.79 ± 0.25
Fangchinoline	*y* = 7435.7*x* − 4899.2	0.9990	3.125–100	5.03 ± 0.33
Tetrandrine	*y* = 6608.7*x* − 15895	0.9996	6.25–200	11.14 ± 0.73

The linearity curves were constructed by plotting the peak area versus the concentration of each alkaloid. Each regression equation was derived from six data points (*n* = 6). For each alkaloid, the correlation coefficient *R*^2^ was >0.999, indicating a good linear relationship between peak area and concentration. The main alkaloids in STR crude drug were determined (*n* = 3).

**Table 2:** The binding residues of fangchinoline and cyclanoline with the AChE active pocket.

Name	Alkaloid Binding Interactions with AChE					Scores ^1^
	Receptor	Ligand	Interaction	Binding Affinity kJ/mol	Active Site	
Fangchinoline	Trp-286	6-ring (B, C′)	π–π	−35.14	PAS ^2^	−7.11 ± 0.32
		H (1-CH; 3, 9, 11′-CH_2_; 11, 14-Ar H)	H–π	−35.85		
	Leu-76	6-ring (C′)	π–H	−2.74	/	
	Leu-289	6-ring (C)	π–H	−3.34	/	
	Val-294	6-ring (C)	π–H	−0.70	/	
	Tyr-341	6-ring (C′)	π–H	−2.41	PAS	
	Glu-292	H (7′-OH)	H–donor	−5.68	/	
Cyclanoline	Trp-286	6-ring (A, D)	π–π	−33.24	PAS	−7.00 ± 0.01
		H (1-Ar H; 5, 8-CH_2_; 10-OCH_3_)	H–π	−45.78		
	Trp-86	H (4-Ar H; 10-OCH_3_)	H–π	−4.92	AS ^3^	
	Ser-293	H (6, 8-CH_2_; 7-NCH_3_; 9-OH)	H–donor	−10.99	/	
	Tyr-341	H (3, 10-OCH_3_)	H–π	−1.95	PAS	
	Asp-74	N (7)	Metal/ion	−2.64	PAS	
		H (2, 9-OH), H (8-CH_2_; 14-CH)	H–donor	−8.95		
	Glu-202	H (9-OH)	H–donor	−3.73	/	
	His-447	6-ring (D)	π–H	−1.65	CS ^4^	
		H (2-OH)	H–donor	−1.33		

The binding modes of fangchinoline and cyclanoline with AChE were analyzed by summarizing the interactions between the ligand and AChE residues. ^1^ The score is expressed as the average of three independent fitting scores (mean ± SEM), and lower scores indicate a better fitting effect. ^2^ PAS represents the peripheral anionic site, ^3^ AS represents the anionic site, ^4^ CS represents the catalytic site, and “/” represents an unknown site.
